# Remifentanil Protects Human Keratinocytes against Hypoxia–Reoxygenation Injury through Activation of Autophagy

**DOI:** 10.1371/journal.pone.0116982

**Published:** 2015-01-23

**Authors:** Jae-Young Kwon, Bong-Soo Park, Yong-Ho Kim, Yong-Deok Kim, Cheul- Hong Kim, Ji-Young Yoon, Ji-Uk Yoon

**Affiliations:** 1 Department of Anesthesia and Pain Medicine, School of Medicine, Pusan National University, Yangsan, Korea; 2 Department of Oral Anatomy, School of Dentistry, Pusan National University, Yangsan, Korea; 3 Department of Oral and Maxillofacial Surgery, School of Dentistry, Pusan National University, Yangsan, Korea; 4 Department of Dental Anesthesia and Pain Medicine, School of Dentistry, Pusan National University, Yangsan, Korea; IISER-TVM, INDIA

## Abstract

The proliferation, differentiation, and migration of keratinocytes are essential in the early stages of wound healing. Hypoxia-Reoxygenation (H/R) injury to keratinocytes can occur in various stressful environments such as surgery, trauma, and various forms of ulcers. The effects of remifentanil on human keratinocytes under hypoxia-reoxygenation have not been fully studied. Therefore, we investigated the effects of remifentanil on the proliferation, apoptosis, and autophagic activation of human keratinocytes during hypoxic-reoxygenation. Human keratinocytes were cultured under 1% oxygen tension for 24 h. The cells were then treated with various concentrations of remifentanil (0.01, 0.1, 0.5, and 1 ng/mL) for 2 h. Thereafter, the cells were reoxygenated for 12 h at 37°C. We measured cell viability via MTT assay. Using quantitative real-time PCR and western blot analysis, we measured the expression levels of proteins associated with apoptosis and autophagy. Quantification of apoptotic cells was performed using flow cytometer analysis and autophagic vacuoles were observed under a fluorescence microscope. Remifentanil treatment brought about an increase in the proliferation of human keratinocytes damaged by hypoxia-reoxygenation and decreased the apoptotic cell death, enhancing autophagic activity. However, the autophagy pathway inhibitor 3-MA inhibited the protective effect of remifentanil in hypoxia-reoxygenation injury. In conclusion, the current study demonstrated that remifentanil treatment stimulated autophagy and reduced apoptotic cell death in a hypoxia-reoxygenation model of human keratinocytes. Our results provide additional insights into the relationship between apoptosis and autophagy.

## Introduction

Wound healing is important not only for repairing the skin but also for its beneficial effects on systemic physiological defenses. Re-epithelialization initiated during the early stages of healing is a critical factor in wound healing. This process includes the proliferation, differentiation, and migration of keratinocytes to the wound margins [1,2].

Ischemia–reperfusion in skin can occur during various stressful environments such as surgery and trauma. Pressure, diabetic, and varicose ulcers also can give rise to hypoxia-reoxygenation injury of keratinocytes [3–5].

Apoptosis has a major role in the elimination of inflammatory cells and formation of granulation tissue in the normal wound healing process. Apoptosis and autophagy in keratinocytes are inevitable during ischemia-reperfusion, induced by skin injury [6].

Autophagy is a self-eating process that is important for balancing sources of energy at critical times during development and in the responses to stress. Autophagy also plays a crucial role in removing damaged intracellular organelles and aggregated proteins as well as eliminating intracellular pathogens [6–8].

Remifentanil, an ultra-short-acting mu-opioid receptor agonist, is unique because of its esterase-based metabolism, minimal accumulation, and very rapid onset and offset of clinical action. Remifentanil prevents the inflammatory response and suppressed inducible nitric oxide synthase expression in septic mouse models [9]. After cardiopulmonary bypass coronary artery surgery, remifentanil inhibited the release of biomarkers of myocardial damage [10]. However, the effects of remifentanil on human keratinocytes and autophagy during hypoxia-reoxygenation are yet to be fully studied. Here, we investigated the protective effect remifentanil confers against hypoxia-reoxygenation injury in human keratinocytes and whether this effect is mediated by autophagy.

## Materials and Methods

### 1. Cell culture

Human keratinocytes (HaCaT cell line) were obtained from the American Type Culture Collection (ATCC, Manassas,USA). Dulbecco's modified Eagle's medium (DMEM, GIBCO) supplemented with 10% inactivated fetal bovine serum (FBS, GIBCO) containing 500 µg/mL penicillin and 500 µg/mL streptomycin (GIBCO), was used to culture the cells at 37°C in a humidified environment with 5% CO_2_. Media were changed once every 3 days.

### 2. Hypoxia/reoxygenation of cultured human keratinocytes and drug treatment

**Figure 1:**
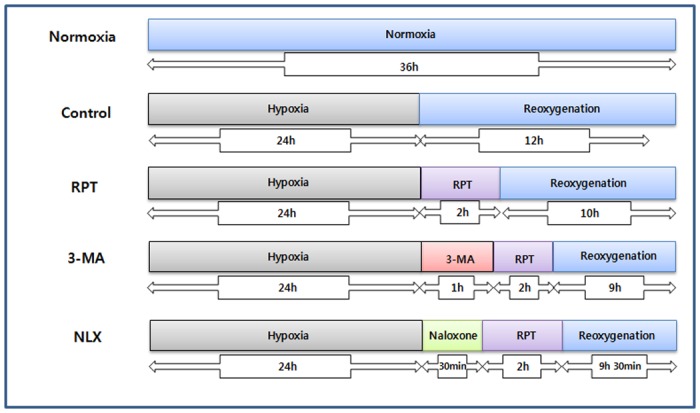
The experimental protocols followed for all *in vitro* experiments are represented as follows. Normoxia = normoxia group, Control = no remifentanil treatment group, RPT = remifentanil post treatment group, 3-MA = both 3-MA and remifentanil treatment group, NLX = both naloxone and remifentanil treatment group.

The human keratinocytes were seeded in a 96-well plate (1 × 10^4^ cells) before exposure to hypoxia-reoxygenation. The cells were gassed with 94% N_2_, 5% CO_2_ and 1% O_2_ incubated for 24 h at 37°C. After hypoxic condition, they were rapidly transferred into a normoxic incubator with DMEM for reoxygenation. To determine whether the administration of remifentanil (Ultiva; Glaxo Smith Kline Pharmaceuticals,UK) affects human keratinocytes in hypoxia-reoxygenation injury, cells which were taken out from the hypoxic incubator were immediately exposed to various concentrations of remifentanil (0.01, 0.1, 0.5, and 1 ng/mL) and normoxia for 2 h. After remifentanil treatment, to simulate reoxygenation and recovery, the cells were reoxygenated for 10 h at 37°C. Control group did not receive remifentanil treatment. Normoxia group did not receive hypoxia or remifentanil treatment for 36 h. The 3-MA group was treated with 3-methyladenine (3-MA) for 1 h before remifentanil treatment and the NLX group was treated with naloxone for 30 min before remifentanil treatment. (Fig. 1)

### 3. MTT assay

The viability of cultured cells was estimated by MTT assay (MTT, Amresco). In the MTT assay, cells were placed in a 96-well plate and incubated for 24 h. After remifentanil treatment, the cells were treated with 0.5 mg/ml of MTT in growth medium. Cell viability was measured using an ELISA reader (Quant, Bio-Tek, Highland Park, USA) at 570 nm.

### 4. Flow cytometer analysis (with 1 ng/mL remifentanil treatment)

Measurement of apoptotic HaCaT cells was determined by Annexin V-FITC/PI staining. In each group, the harvested cells were washed with PBS containing 1% bovine serum albumin and centrifuged at 2,000 rpm for 10 min. The cells were resuspended in ice-cold 95% ethanol with 0.5% Tween 20. The fixed cells were washed in 1% BSA-PBS solution and then stained with 5 μL Annexin V–FITC and 5 μL propidium iodide (PI) (50 μg/mL). The results were expressed as quadrant dot plots with intact phase (Annextin V-/PI-), early apoptotic phase (Annextin V+/PI-), late apoptotic phase (Annextin V+/PI+) and necrotic phase (Annextin V-/PI+). Mean expression of apoptotic and necrotic HaCaT cells were analyzed by flow cytometer. The number of cells in each quadrant was expressed as percentages of total stained cells [11].

### 5. Hoechst staining (with 1 ng/mL remifentanil treatment)

After remifentanil pretreated, the cells were harvested onto a clean, fat-free glass slide with a cytocentrifuge. They were stained in 4 μg/mL Hoechst 33342 for 10 min at 37°C in darkness and washed twice in PBS. The slides were mounted with glycerol. The samples were observed and photographed using epifluorescence microscopy (Carl Zeiss, Göettingen, Germany). The number of cells showing condensed or fragmented nuclei was determined by a blinded observer from a random sampling of 3 × 10^2^ cells per experiment. Three independent experiments were conducted.

### 6. Fluorescence microscopy (with 1 ng/mL remifentanil treatment)

Cells were grown on cover slips and treated with HS-1200. After 24 h, cells were stained with 0.05 mM MDC, a selective fluorescent marker for autophagic vacuoles, at 37°C for 1 h. The changes in cellular fluorescence were observed using a microscope (Axioskop, Carl Zeiss, Germany). For further detection of the acidic cellular compartment, we used acridine orange, which emits bright red fluorescence in acidic vesicles but emits green fluorescence in the cytoplasm and nucleus. Cells were stained with 1 μg/mL acridine orange for 15 min and washed with PBS. AVOs formation was observed using a LSM 700 confocal microscope (Carl Zeiss, Germany).

### 7. Western blot analysis (with 1 ng/mL remifentanil treatment)

Cells were plated at a density of 2 x 10^6^cells in 100 mm culture dishes. Cells treated with remifentanil were washed twice with ice-cold PBS and centrifuged at 2,000 rpm for 10 min.

Total cell proteins were lysed with a RIPA buffer [300 mMNaCl, 50 mM Tris-HCl (Ph 7.6), 0.5% Triton100, 2Mm PMSF, 2 μg/ml aprotinin and 2 μg/ml leupeptin] and incubated at 4°C for 30 min. The lysates were centrifuged at 14,000 rpm revolutions per min for 15 min at 4°C, and sodium dodecyl sulfate (SDS) and sodium deoxycholicacid(0.2% final concentration) were added. Protein concentrations of cell lysates were determined with Bradford protein assay (Bio-Rad Richmond,CA USA) and BSA was used as a protein standard. A sample of 20 μg protein in each well was separated and it was loaded onto 7.5–15% SDS/PAGE.

The gels were transferred to Nitrocellulose membrane (Amersham GE Healthcare, Little Chalfont, UK) and reacted with each antibody. Immunostaining with antibodes was performed using Super Signal West Femto enhanced chemiluminescence substrate and detected with Alpha Imager HP (Alpha Innotech, Santa Clara, USA). Equivalent protein loading was confirmed by Ponceau S staining. Antibodies used in the study were cleaved caspase-3 (1:1,000), cleaved caspase-9 (1:1,000), Bcl-xl (1:1,000), Bax (1:1,000), LC3 (1:3,000), Becline-1 (1:1,000) from Abcam and P62(1:1,000) and Atg5 (1:500) from Santa Cruz.

### 8. In vitro wound healing (with 1 ng/mL remifentanil treatment)

Cells were seeded into a cover glass for microscope slides in DMEM with 10% FBS. The confluent cell basal was scratched with an Eppendorf sterile pipette tip to steadily produce a circular cell-free zone (1 mm in diameter) on the coverslips. The wound cultures were then incubated at 37°C for 24 h, and wound healing process was observed under optical microscopy. After 24 h incubation in growth medium at 37°C, the new growth and migration into the cell-free zone was considered as the process of *in vitro* healing. Images of the relatively healed monolayer area were obtained and the pixel counts were analyzed using NIH Image J software.

### Statistical analysis

All experiments were repeated five times. Multiple groups were compared using one-way analysis of variance (ANOVA) followed by a post hoc Turkey test. The data were expressed as mean ± standard deviation (SD). P < 0.05 was considered as significant score (SPSS 13.0 Software, SPSS Inc., Chicago, IL, USA).

## Results

### Effect of remifentanil treatment on cell viability

**Figure 2:**
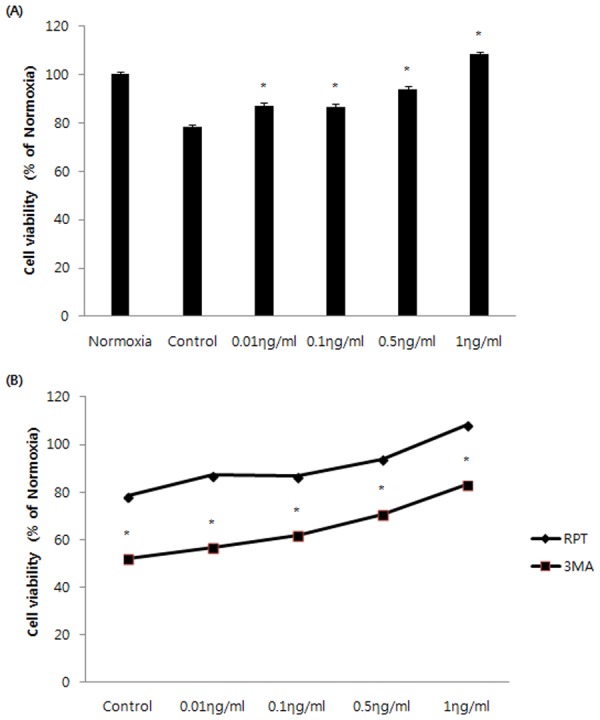
The effect of remifentanil on cell viability in human keratinocytes assessed by MTT assay. (A) Effect of remifentanil on HaCaT cells under H/R conditions assessed by MTT assay. *P < 0.05 as compared with the control group.(B) Cell viability comparison between RPT and 3MA groups.*P < 0.05 as compared with the RPT group.

The cell viability rate (%) was calculated and compared with the control group. Control group had lower cell viability than normoxia group. However, the cell viability was higher in the 0.01, 0.1, 0.5, and 1 ng/mL groups compared to the control group (P <0.05, Fig. 2A). In 3-MA group, the cell viability was lower than that of the remifentanil groups (P <0.05, Fig. 2B).

Among all the concentrations, the cell viability was highest at 1 ng/mL remifentanil. Based on this result, all subsequent experiments were performed with 1 ng/mL remifentanil.

### Effects of remifentanil treatment on apoptosis

**Figure 3:**
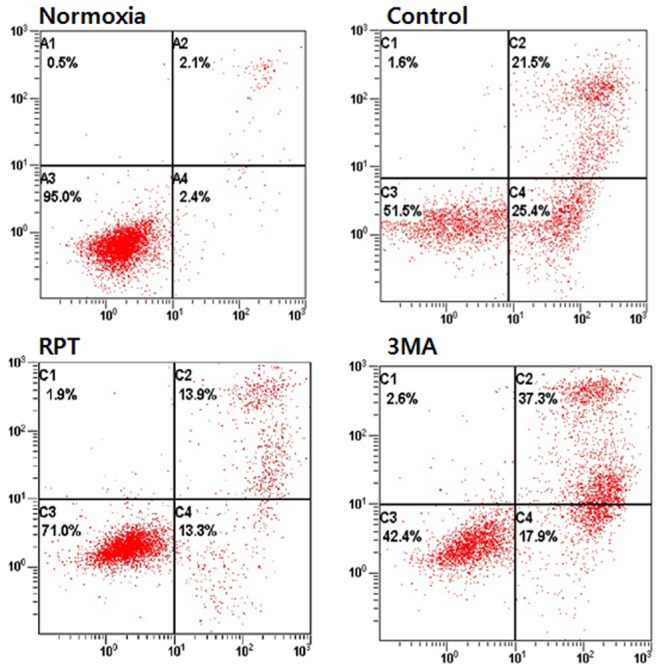
Detection of apoptosis and necrosis with Annexin-V-FITC and propidium iodide staining. All the groups of cells with Annexin-V and propidium iodide staining were measured by flow cytometry. Normoxia = normoxia group, Control = no remifentanil treatment group, RPT = remifentanil post treatment group, 3-MA = both 3-MA and remifentanil treatment group.

**Figure 4:**
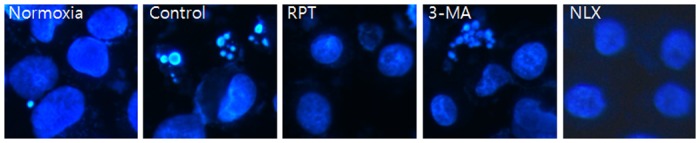
Hoechst staining: Morphological changes in H/R-induced HaCaT cells treated with remifentanil (1ng/ml), 3-MA and naloxone. H/R-induced HaCaT cells treated with remifentanil, 3-MA and naloxone as observed by fluorescence microscopy. Apoptotic bodies were seen in control and 3-MA group cells. In contrast they were markedly reduced in RPT and NLX group cells. Normoxia = normoxia group, Control = no remifentanil treatment group, RPT = remifentanil post treatment group, 3-MA = both 3-MA and remifentanil treatment group, NLX = both naloxone and remifentanil treatment group.

**Figure 5:**
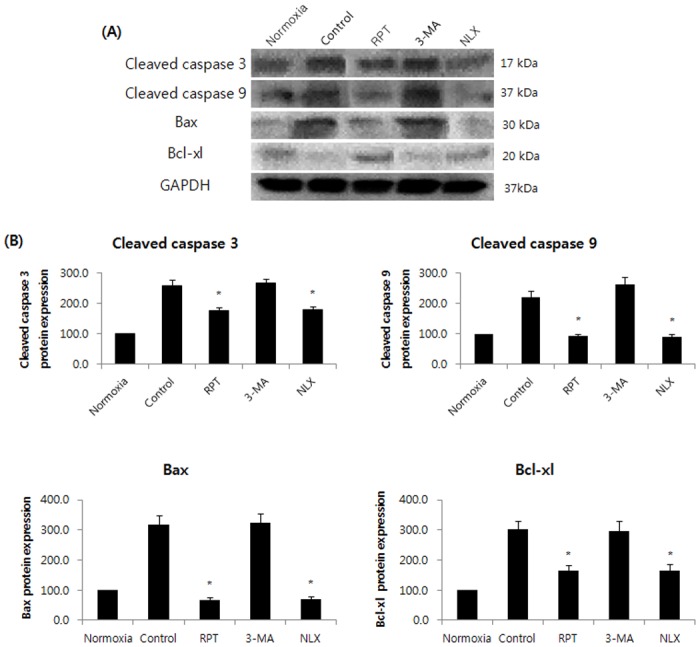
Effects of remifentanil on the expression of caspase-3, caspase-9, Bcl-xl, and Bax in human keratinocytes. Western blot analysis and densitometry. In RPT and NLX groups, cellular expression of cleaved caspase-3, 9, and BAX were down-regulated while that of Bcl-xl was elevated. *P < 0.05 as compared with the control group.

Annexin-V FITC/PI staining confirmed the anti-apoptotic effects of remifentanil quantitatively (Fig. 3). The portion of annexin-V(+) cells in H/R and 3-MA groups increased by 46.9% and 55.2% (P < 0.05). However, remifentanil treatment significantly attenuated the percentage of annexin-V(+) cells to 27.2% (P < 0.05), demonstrating the anti-apoptotic effect of remifentanil.To ascertain the effect of remifentanil on apoptosis, Hoechst 33258 staining was used to detect the cells under a fluorescence microscope (×400, Fig. 4). A majority of the cells in normoxia, RPT and NLX groups showed normal morphology with round regular nuclei. In contrast, apoptotic bodies were seen in control group and 3-MA group cells. However, remifentanil treatment effectively prevented the apoptosis of cells as indicated by the morphological analysis and the protective effect was not blocked by naloxone treatment. We also examined the expression of factors associated with apoptosis in the cells subjected to H/R by western blotting analysis. In RPT and NLX groups, cellular expression of cleaved caspase-3, 9, and BAX were down-regulated while that of Bcl-xl was elevated (Fig. 5).

### Effect of remifentanil treatment on the activation of autophagy

**Figure 6:**
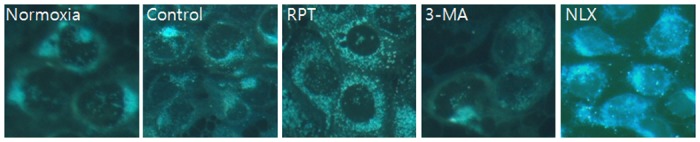
MDC staining of cytoplasmic vacuoles after remifentanil treatment in human keratinocytes. Fluorescence microscopic (×400) analysis of autophagosome in the H/R injured HaCaT cells. H/R caused accumulation of autophagosomes containing partially digested cytoplasmic contents compared to the control group. The remifentanilduring H/R dramatically increased formation of autophagosomes and the autophagy pathway inhibitor 3-MA blocked formation of autophagosomes by remifentanil. But naloxone did not block that.

**Figure 7:**
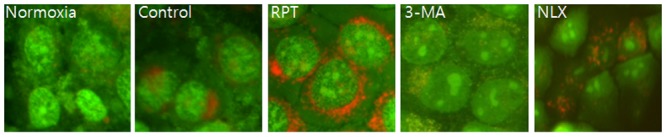
AO staining of autophagosomes after remifentanil treatment in human keratinocytes. Fluorescence microscopic (×400) analysis of autophagosome in the H/R injured HaCaT cells. Stained with acridine orange the green shows where the dye has stained the nucleus and the red is where the cell is starting to’digest' parts of itself in small capsules called autophagasomes. H/R caused accumulation of autophagosomes containing partially digested cytoplasmic contents compared to the control group. The remifentanil during H/R dramatically increased formation of autophagosomes and the autophagy pathway inhibitor 3-MA blocked formation of autophagosomes by remifentanil. But naloxone did not block that.

**Figure 8:**
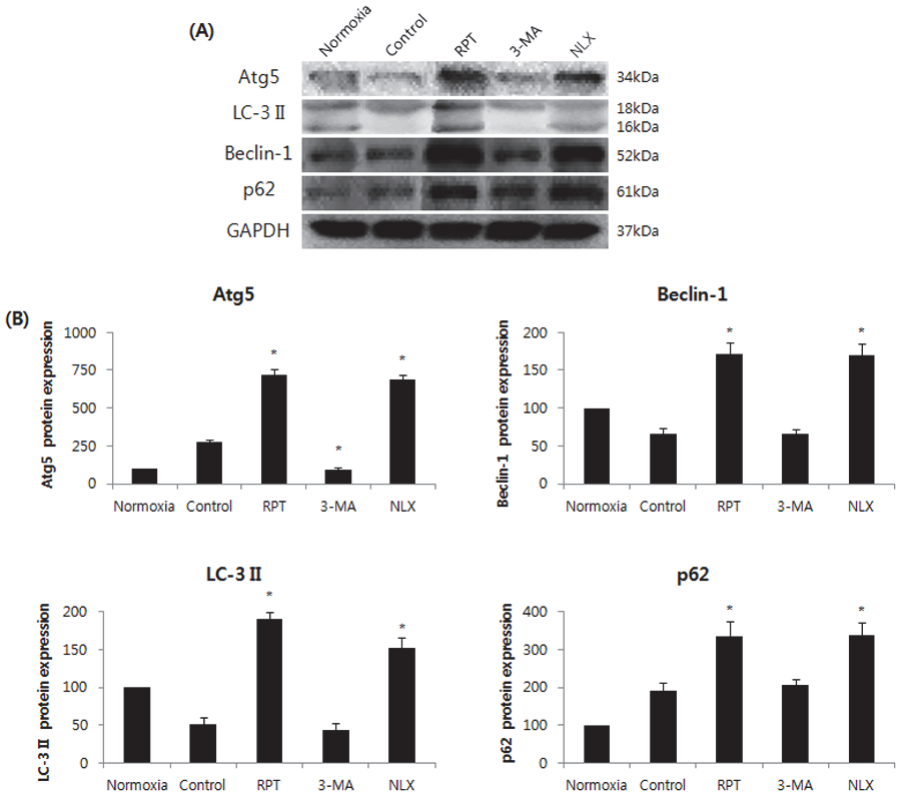
Effects of remifentanil on autophagy markers in human keratinocytes. Western blot analysis and densitometry. ATG5, Becline-1, LC-3 II, and P62 were elevated in RPT and naloxone group cells. *P < 0.05 as compared with the control group.

Significant accumulation of autophagic specific staining of MDC was observed around the nuclei in RPT and NLX groups cells (Fig. 6). Similarly, AO staining, indicated by red fluorescent spots appeared in RPT and NLXgroups cells, while the normoxia, control, and 3-MA groups showed mainly green cytoplasmic fluorescence (Fig. 7). We examined the activation of autophagy related proteins in H/R-induced cells by western blotting analysis. ATG5, Becline-1, LC-3 II, and P62 were elevated in RPT and NLX groups. However, the protein levels decreased when autophagy was suppressed by 3-MA (Fig. 8).

### Effects of remifentanil treatmenton cell migration

**Figure 9:**
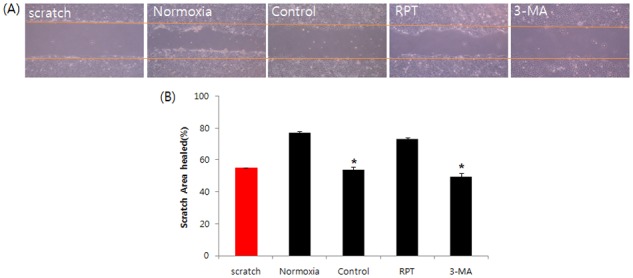
*In vitro* wound healing. Remifentanil restored cell proliferation and migration, which had been decreased by hypoxia. Investigation of cell migration capability after H/R was performed.Confluent monolayers of HaCaT cells were wounded by scratching the surface as uniformly as possible with a 1 mL pipette tip. *P < 0.05 as compared with the normoxia group.

The *in vitro* model of wound healing was used to examine the effects of remifentanil on wound healing(Fig. 9). In normoxia group, cells rapidly migrated to close the wound after 24 h. However, in the control group and 3-MA group, there was a significant delay in healing (*p* < 0.05), with decreased density. Cells treated with remifentanil exhibited faster migration into the wounded area compared to the control group and 3-MA group cells (*p* < 0.05).

## Discussion

The object of the current study is to determine the beneficial effect of remifentanil on human keratinocytes in hypoxia-reoxygenation injury and to investigate whether autophagy is associated with the protective mechanism. This study provides four principal findings. First, we showed that remifentanil treatment increased the proliferation of human keratinocytes in hypoxia-reoxygenation injury (Fig. 2A). Second, remifentanil treatment protected human keratinocytes against hypoxia-reoxygenation induced apoptosis. Using western blot analysis, we showed that remifentanil treatment decreased cleaved caspase-3,-9 levels, related to caspase-dependent pathway, and reduced Bax/Bcl-xl ratio, which is associated with mitochondrial related pathway. Activation of the upstream regulators cleaved-caspase-3 and-9 is a key to the initiation and induction of apoptosis [12,13]. It is known that the mitochondria-dependent apoptotic pathway is regulated by Bcl-xl protein family, such as the anti-apoptotic protein Bcl-xl and pro-apoptotic protein Bax, which are critical downstream regulators in caspase activation [14,15].

Third, the anti-apoptotic effect of remifentanil treatment in human keratinocytes was probably due to the induction of intracellular autophagy. The intracellular autophagy pathway inhibitor 3-MA blocked the protective effect of remifentanil treatment on cellular apoptosis, suggesting a key role for intracellular autophagy in remifentanil treatment. In autophagy-specific staining of MDC and AO, RPT group demonstrated more autophagic expression than normoxia, control, and 3-MA groups. In the western blot analysis, we showed that remifentanil treatment increased the expression of ATG5, Beclin-1, LC-3 II, and P62 proteins associated with autophagy. We also demonstrated that protective effectthrough activation of autophagy byremifentanil was not blocked by naloxone. This result suggests that an opioid-receptor signaling does not mediate this effect.

ATG5 induces autophagy and is essential for autophagosome formation [16]. Beclin1 regulates the kinase activity in the activation of mammalian Vps34, which is an initial step in vesicle nucleation [17,18]. LC3-II is used as anautophagy marker because its lipidation and specific recruitment of autophagosomes results in a shift from diffuse to punctate staining of the protein and increases its electrophoretic mobility on gels compared with LC3-I [19]. p62/SQSTM1 is a multifunctional adaptor protein that promotes the turnover of polyubiquitinated protein aggregates through interaction with LC3 at the autophagosome [20,21].

Fourth, a wound-healing assay was performed to determine the migratory capability of human keratinocytes. RPT group showed increased wound healing capability. Migration of keratinocytes is a mandatory step in wound healing [22]. Diminished healing of keratinocytes might lead to defective wound healing. The interaction between fibroblasts and keratinocytes is important during wound healing. The expression of collagen can affect cell migration and adhesion, and stimulate the metabolism of connective tissues [23].

Based on these results, we suggest that remifentanil treatment stimulated the endogenous cellular protective effect in human keratinocytes against hypoxia-reoxygenation injury through the activation of signaling pathways associated with autophagy. Previous studies have reported the effect of autophagy against hypoxia-reoxygenation injury in other cells, but the effect of remifentanil on autophagy in human keratinocytes with hypoxia-reoxygenation injury has not been documented.

## Conclusions

The present study shows that remifentanil treatment increases the human keratinocyte proliferation rate and stimulates the expression of autophagy under hypoxia-reoxygenation injury. No functional studies were performed to investigate the effects of remifentanil on wound healing process. Our results suggest that remifentanil may have a beneficial effect in the recovery of wounds caused by hypoxia-reoxygenation injury.
